# ^1^H -NMR Metabolomics Study of the Effect of Cisplatin and Casiopeina IIgly on MDA-MB-231 Breast Tumor Cells

**DOI:** 10.3389/fmolb.2021.742859

**Published:** 2021-12-01

**Authors:** Karen Resendiz-Acevedo, Martha E. García-Aguilera, Nuria Esturau-Escofet, Lena Ruiz-Azuara

**Affiliations:** ^1^ Facultad de Química, Universidad Nacional Autónoma de México, Ciudad de México, Mexico; ^2^ Instituto de Química, Universidad Nacional Autónoma de México, Ciudad de México, Mexico

**Keywords:** breast carcinoma, metallodrug, 1 H-NMR metabolomics, Casiopeinas ^®^, Copper

## Abstract

The knowledge of the metabolic processes of designed metallodrugs for cancer treatment is an area that has been not profoundly studied. Casiopeina IIgly (CasIIgly), which belongs to the Casiopeínas^®^ family, is a copper (II) coordination compound that has shown good biological activity against several cancer cells, low toxicity in normal cells, and antineoplastic activity in *in vivo* murine and xenografted models. In this work we employed a triple-negative highly metastatic breast carcinoma line (MDA-MB-231), which is one of the cancer types with a great mortality index, for ^1^H-NMR metabolomic analysis using cisplatin and CasIIgly, in order to quantify the effect of metallodrugs in the metabolic profile of this cell tumor line as a consequence of treatment at different times. Our findings indicate that cisplatin mainly contributes to phospholipid biosynthesis while CasIIgly affects processes such as carbohydrates and nucleotides metabolism. Also, we observed that CasIIgly treatment has an important and fast effect over MDA-MB-231 cell metabolism, which makes it a good alternative for treatment in this type of cancer.

## Introduction

When cancer cells transform into the malignant variant, they acquire new characteristics such as unlimited transformation, sustained angiogenesis, loss of apoptosis, tissue invasion, metastasis, and metabolic reprogramming, among others (Hanahan and Weinberg, 2000; Romero-Garcia et al., 2011; Kalyanaraman, 2017). Metabolic reprogramming provides resources for maintaining ATP, NADH, and NADPH levels for cell survival. These modifications are the result of intrinsic and extrinsic factors such as oncogenic signaling and tumoral microenvironment (Romero-Garcia et al., 2011; Jeon and Hay, 2018).

Glycolysis, glutaminolysis, and fatty acids synthesis are some important modified pathways in cancer. Glycolysis is the fundamental process for energy generation where the principal substrate is glucose. Otto Warburg observed that in cancer cells, this pathway favors lactate formation, and a small part of glucose is used for ATP generation, this processes modification was called the “Warburg effect” (Romero-Garcia et al., 2011; Zheng, 2012; Liberti and Locasale, 2016). On the other hand, glutaminolysis involves different biochemical reactions where glutamine is converted in glutamate and alfa-ketoglutarate, which act as intermediaries for electron transport chains such as NADH and FADH_2_, as well as carbon and nitrogen used in amino acid synthesis (Zhang and Ming, 2013; Yang et al., 2017).

Lipid metabolism involves biological molecules which are constituents of membrane cells and are also responsible for energetic reserves. In cancer, this process is upregulated and it is associated with metastasis and carcinogenesis phenomena (Yang et al., 2017; Cheng et al., 2018; Long et al., 2018; Ocaña et al., 2020).

In summary, these metabolic pathways are related to tumor cell progression, proliferation, cell survival, apoptosis, cell morphology, cell adhesion, cell motility, angiogenesis, and maintenance of the tumor (Yang et al., 2018). For these reasons, it is important to search for new more effective and less toxic alternatives for disease treatment. The Casiopeinas® are coordination compounds which have copper as their central atom, they substitute bipyridine or phenanthroline as the primary ligand and glycinate or acetylacetonate as the secondary ligand (Ruiz-Azuara, 1990; Ruiz-Azuara, 1992; Ruiz-Azuara, 1996; Ruiz-Azuara, 1997). These compounds have been shown to be biologically active in diverse tumor cells *in vitro* as well as *in vivo* (Trejo et al., 2005; Carvallo-Chaigneau et al., 2008; Bravo-Gómez et al., 2009; Ruiz-Azuara and Bravo, 2010; Bravo et al., 2013), and it is important to mention that the Casiopeinas® present a selectivity to tumor cells as their effect over healthy cells is low (Espinal-Enríquez et al., 2016; García-Ramos et al., 2017).

Their action mechanisms involve DNA interaction, generation of reactive oxygen species (ROS), apoptosis induction, and it was also observed that they have effects over cancer cell metabolism processes such as oxidative phosphorylation and over hexokinase II enzyme activity, which is related to glycolysis (Marín-Hernández et al., 2012; de Anda-Jáuregui et al., 2019).

It was observed in previous works that the Casiopeinas® can produce deregulation in mitochondrial function in cancer cells at 6 h after drug exposition, as well as apoptosis and autophagia processes at 2 and 10 h, respectively (Valencia-Cruz et al., 2013; García et al., 2017; Vázquez-Aguirre et al., 2019).

In this work, we describe the results of the MDA-MB-231 ^1^H-NMR metabolic profile when these cells are treated with cisplatin [diamin, dichloride platinum (II)] and Casiopeina IIgly [Cu 4,7-dimethyl 1,10-fenanthroline, glycinate copper (II)] (CasIIgly) after 20 and 40 min.

## Materials and Methods

### Cell Culture

In this study, we used MDA-MB-231 (hormonal-independent triple-negative breast cancer). Cells were cultured in DMEM-F12 (Biowest), consisting of 10% fetal bovine serum (Biowest), 1% antibiotic-antimycotic (ATCC), and 0.5% non-essential amino acids (GIBCO) in a humidified incubator at 37°C, in the presence of 5% CO_2_. When the mixture had 80% confluency, the cells were separated with Versene (KCl, EDTA, NaCl, and Tris-HCl), and cell viability was determined through a Trypan blue assay (4%) with a Neubauer camera (Strober, 2001).

### Chemical and Reagent

NaH_2_PO_4_, Na_2_HPO_4_, deuterium oxide (D_2_O) (D, 99.98%), and cisplatin were purchased from Sigma-Aldrich (St. Louis, MO, United States). Trimethylsilyl propionic acid-d_4_ sodium salt (TSP) was supplied by Merck (Darmstadt, Germany). CasIIgly (4,7-dimethyl, 1,10-phenanthroline, glycinato copper (II) nitrate, monohydrate) was synthetized as in patents (Ruiz-Azuara, 1990; Ruiz-Azuara, 1996; Ruiz-Azuara, 1997).

### Determination of the Mean Inhibitory Concentration by MTT Assay

The cells were cultured in 96-well plates with 10,000 cells/well with 100 μL of medium in a humidified incubator at 37°C, in the presence of 5% CO_2_ for 24 h. Subsequently, the culture medium was withdrawn and 90 μL of medium and 10 μL of compound solution was added. Cells were exposed to treatment for 24 h and after this were retired. Then, an MTT assay was conducted according to the procedure reported by Mosmann (Mosmann, 1983).

### Cell Culture and Metabolite Extraction

A total of 1,000,000 cells were cultured in Petri dishes (10 cm) in a humidified incubator at 37°C, in the presence of 5% CO_2_, and 5.4 ml of medium was added with 600 μL of compound solution in triplicate during the evaluation times (20 and 40 min, without treatment, cisplatin, and CasIIgly). After the treatment time, the cells were separated and again their viability was checked by a Trypan blue assay. For metabolite extraction, the cell suspension was centrifuged at 3,500 × g for 5 min at 4°C, the supernatant was discarded, and the cell button was maintained for 5 min in ice. Subsequently, 1 ml of acetonitrile/water mixture (50%) was added and the suspension was sonicated for 20 min. Finally, the cells were centrifuged at 14,500 × g for 15 min at 4°C. The supernatant was recovered and the acetonitrile was evaporated (Beckonert et al., 2007).

The dry samples of metabolites was resuspended in 700 μL of PBS-TSP solution [NaH_2_PO4 (1 mM)/Na_2_HPO_4_ (1 mM)/TSP(0.02 mM), pH = 7.4]. The samples were homogenized and centrifuged at 12,000 × g for 5 min. The supernatant of each sample was transferred to a 5-mm NMR tube for analysis.

### ^1^H-NMR Analysis

^1^H-NMR analysis was conducted at 298 K on an Avance III HD spectrometer operating at a ^1^H frequency of 699.95 MHz, 16.4T (Bruker, Billerica, MA, United States) equipped with a 5-mm *z*-axis gradient TCI cryoprobe and SampleJet automatic sample changer. ^1^H-NMR spectrum was recorded using the CPMG (Carr-Purcell-Meiboom-Gill) pulse sequence with residual water signal suppression using presaturation (cpmgpr1d). A total of 256 scans were collected into 64 k datapoints over a spectral width of 8,403.361 Hz, and a relaxation delay of 4 s. Free induction decays (FIDs) were multiplied by an exponential function with a line-broadening factor of 0.3 Hz before Fourier transformation. The ^1^H-NMR spectra were manually corrected for phase and base line distortion using TopSpin 3.5 pl 6 (Bruker, Billerica, MA, United States). ^1^H NMR chemical shifts were referenced to the TSP signal at 0.00 ppm.

### Data Analysis and Quantification of Metabolites

Each ^1^H-NMR spectrum was subdivided into 0.02 ppm buckets and were normalized to the total area. The residual signal of water was excluded. The resulting data matrix were subjected to chemometric analysis in SIMCA 17.0.1 software (Umetrics, Sartorius Stedim Biotech). Principal Component Analysis (PCA) was performed, one of the most useful exploratory and unsupervised techniques to exclude outliers, and to identify the dominant variant in the dataset not associated with the biological effect. Subsequently, Orthogonal Partial Least Squares Discriminant Analysis (OPLS-DA) was used for identifying differences among groups, which uses class memberships to maximize the discrimination between groups for a particular biological effect. The quality of each model was determined by the goodness of fit parameter (*R* ^2^) and the goodness of prediction parameter and the fraction of the total variation predicted by a component (Q^2^). The determination of the metabolites responsible for the separation between control and BD groups was done using the loadings plot and the variable importance in the projection (VIP) value of each bin in the model to identify biomarkers.

The identification and quantification of metabolites were realized with Chenomx NMR Suite v. 8.6 (Chenomx Inc., Edmonton, Canada), based on the location of individual resonances on the spectra using the TSP as internal reference and the area under the curve.

The metabolite concentrations were adjusted for each sample to the cellular volume obtained in their recuperation at the end of the treatment with the compounds for their comparison and analysis.

Quantitative Enrichment Analysis (QEA) was realized with MetaboAnalyst 5.0 software using the metabolites concentration for the identification of the relevant metabolic pathways in each compound analyzing both treatment times together.

## Results

### IC_50_ Determination

The activity of the compounds over the MDA-MB-231 cell line was determined as IC_50_. This concentration for each compound was used for the subsequent experiments. Table 1 shows the CI_50_ for cisplatin and CasIIgly.

**TABLE 1:** CI_50_ obtained for cisplatin and CasIIgly.

Compound	IC_50_ μM
Cisplatin	21.71
CasIIgly	1.55

* Data represent the average of three independent assays.

### Cell’s Viability

The mean inhibitory concentration of the compounds (Cisplatin, 21.71 μM and CasIIgly, 1.55 μM) was administered to the MDA-MB-231 cells for 0, 20, 40, 60, 120 min, and 4 and 6 h, in order to make sure that the effects observed in the metabolomic assay were a result of the treatment and not due to cell death. All cells were maintained above 90% viability even within the compounds, the data are presented in the Supplementary Material.

### Data Analysis and Quantification of Metabolites

Once the viability of the cells was verified, we employed two treatment times: 20 and 40 min for metabolomic experiments as a first approximation for the behavior of these compounds.

**FIGURE 1:**
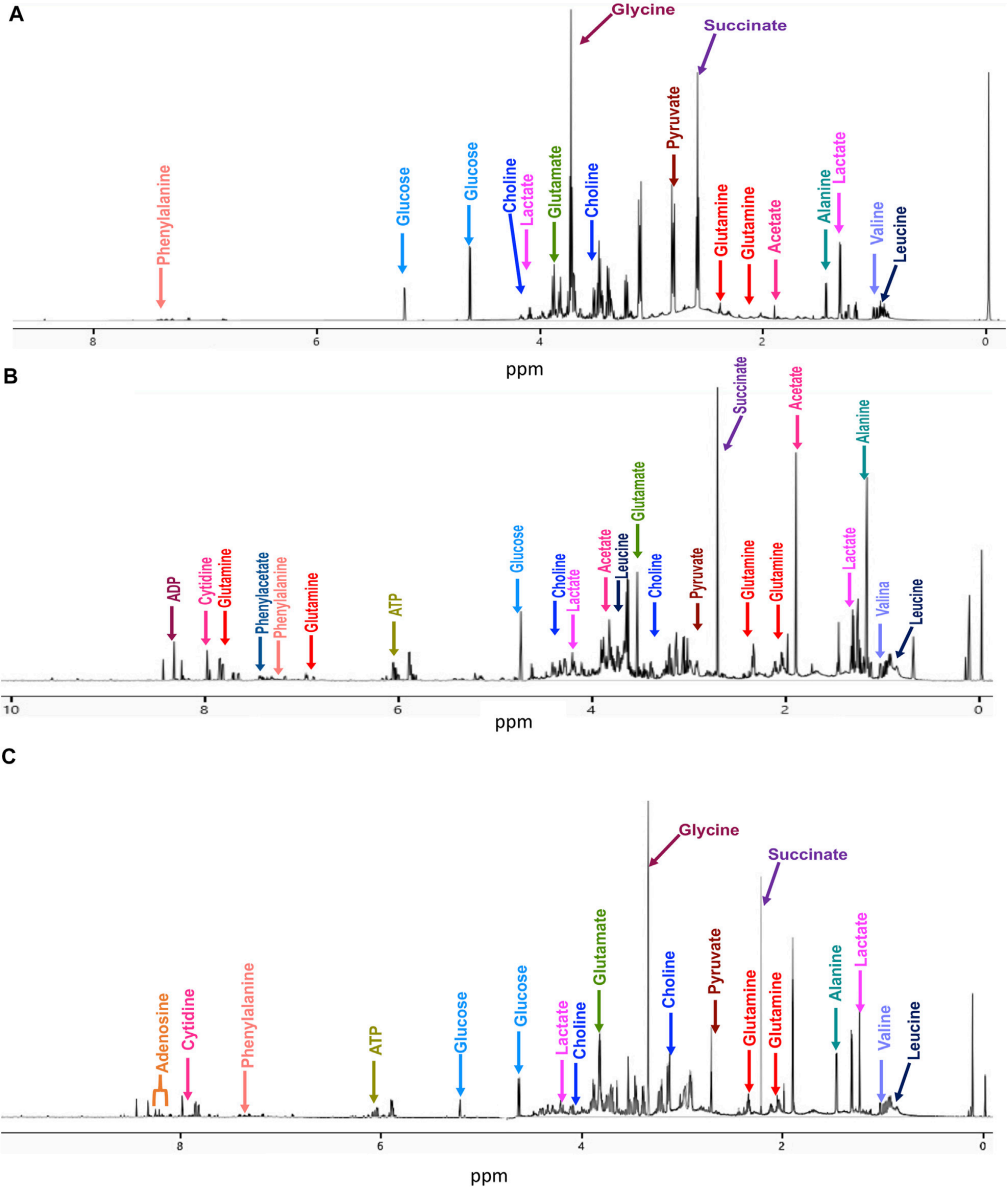
Representative 700 MHz ^1^H-NMR spectra with some metabolite assignments at 20 min treatment for **(A)** untreated cells, **(B)** cisplatin-treated cells, and **(C)** CasIIgly-treated cells.

Typical 700 MHz ^1^H NMR spectra and representative metabolites assignments of the samples are shown in Figure 1.

**FIGURE 2:**
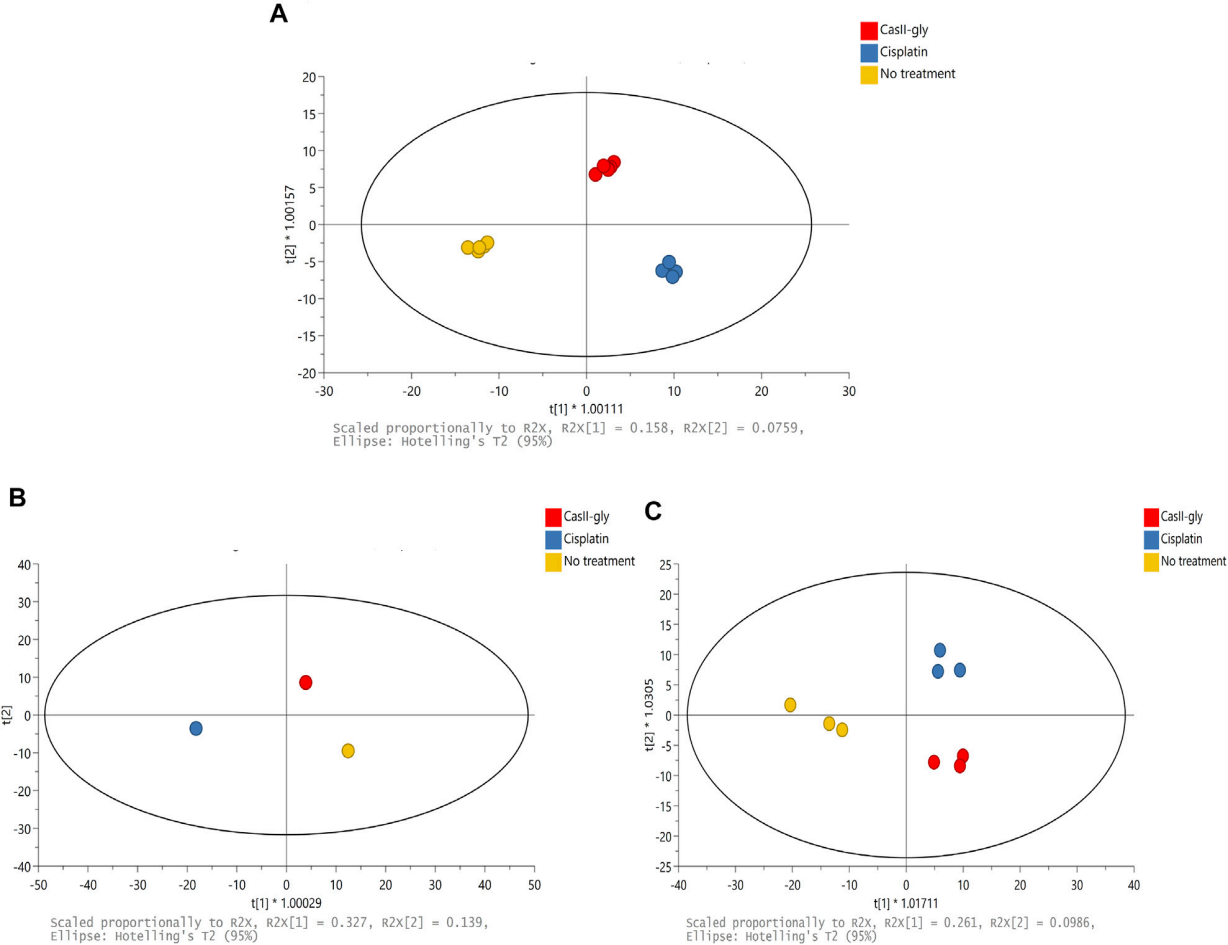
**(A)** Scores of PCA of the ^1^H-NMR spectral data for 20 and 40 min: no treatment (yellow), cisplatin (blue), and CasIIgly (red). **(B)** Scores of OPLS-DA of the ^1^H-NMR spectral data for 20 min: no treatment (yellow), cisplatin (blue), and CasIIgly (red). **(C)** Scores of OPLS-DA of the ^1^H-NMR spectral data for 40 min: no treatment (yellow), cisplatin (blue), and CasIIgly (red).

The ^1^H-NMR spectra of the samples were subjected to multivariate analysis to assess grouping and to identify variations for cells without treatment and with both compounds. Firstly, a supervised method was employed to explore and visualize variations and possible patterns in the dataset (Figure 2A). The resultant model had two latent variables which were able to account for 82% of spectral variation (R^2^X), 97.8% of group variation (R^2^Y), and the prediction accuracy was 72.8% (Q^2^). OPLS-DA showed differences between samples due to the different treatment behaviors. Subsequently, the matrix was divided in two according to the treatment time: one for 20 min and another one for 40 min. For the 20 min treatment, the OPLS-DA had two latent variables which were able to account for 100% of spectral variation (R^2^X), 100% of group variation (R^2^Y), and the prediction accuracy was 100% (Q^2^) (Figure 2B). For the 40 min treatment, the OPLS-DA had two latent variables that were able to account for 80.8% of spectral variation (R^2^X), 89.6% of group variation (R^2^Y), and the prediction accuracy was 57.3% (Q^2^) (Figure 2C). These results suggested that cisplatin and CasIIgly treatments had different and important effects over cell metabolism as they were differently grouped in comparison with no treatment cells even at the same time.

Variable importance in projection (VIP > 1.5) was considered to establish the bins responsible for the separation between samples. Some metabolites assigned to those bins were glucose, choline, alanine, lactate, pyruvate, glutamate, valine, glutamine, leucine, and succinate (Supplementary Tables S1, 2). These metabolites were the cause of the difference between the samples, so they were quantified at both times for each treatment, and each compound treatment was subsequently subjected to QEA, where we considered pathways with a *p*-value <0.05 as statistically significant.

**FIGURE 3:**
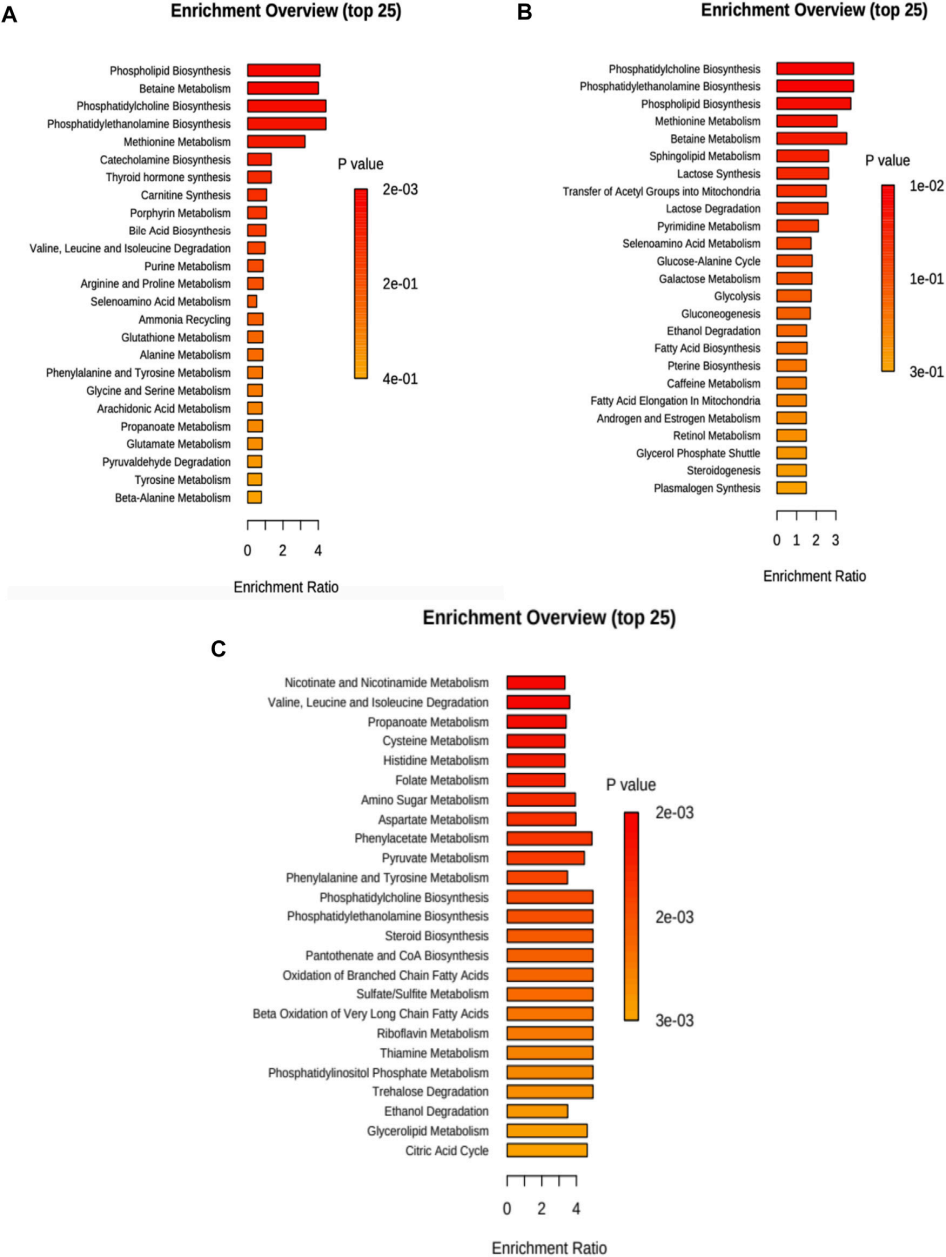
Metabolite set enrichment overview for each case considering 20 and 40 min treatment. **(A)** No treatment, **(B)** cisplatin, and **(C)** CasIIgly. The length of the bar represents the enrichment of the pathway and the color represents the statistical significance (red: mostly significant, orange: less significant).

In Figure 3A (and Supplementary Table S6), we present the altered metabolic routes for no treatment cells. For the discussion section, we only considered metabolic pathways that were statistically significant, and that also had an important effect over cancer progression. With this criterion, phospholipid biosynthesis, betaine metabolism, phosphatidylcholine and phosphatidylethanolamine biosynthesis, and methionine metabolism were relevant.

For cisplatin, the altered metabolic pathways in the samples are presented in Figure 3B (and Supplementary Table S7). For the discussion section, we only considered metabolic pathways that were statistically significant, and that also had an important effect over cancer progression, with this criterion, phosphatidylcholine, phosphatidylethanolamine, phospholipid biosynthesis and methionine metabolism were relevant.

The altered metabolic pathways in the samples due to CasIIgly treatment are shown in Figure 3C (and Supplementary Table S8). For the discussion section, we only considered metabolic pathways that were statistically significant, and that also had an important effect over cancer progression, with this criterion, the Warburg effect, pyruvate metabolism, gluconeogenesis, glycolysis, electron transport chain, *β*-oxidation, and the pentose pathway were relevant.

## Discussion

For no treatment cells, we found important metabolic routes associated with the particular metabolic profile of this type of cancer cell including phospholipid biosynthesis, betaine metabolism, phosphatidylcholine, and phosphatidylethanolamine biosynthesis, as well as methionine metabolism.

Phosphatidylcholine and phosphatidylethanolamine are lipid bilayer components, which help in the metastatic processes in cancer cells, probably for this reason, high lipid concentrations have been found in this type of cell (Gupta et al., 2017; Teng-Hern et al., 2017). On the other hand, betaine and methionine metabolism are altered processes in metastatic cells because they are essential for cell grow (Wanders et al., 2019), then it is important that drugs are capable of delaying processes like these, and in this way retard cancer evolution.

In cisplatin treatment for both times, we found altered metabolic routes such as phosphatidylcholine, phosphatidylethanolamine, phospholipid biosynthesis, and methionine metabolism, which are the same pathways found in no treatment cells, this suggests that the cisplatin treatments can affect metastatic cell proliferation.

Cell treatment with CasIIgly generated a great impact over their metabolism. Although different metabolic routes were found in comparison with no treatment and cisplatin cells, the affectation over metabolism is general and relevant since it was found in essential processes for cell survival such as pyruvate metabolism, gluconeogenesis, glycolysis, electron transport chain, *β*-oxidation, the pentose pathway, and the Warburg effect which are related to alterations in glycolysis due to the fact that in cancer aerobic glycolysis is suppressed and gluconeogenesis significantly participates in this process too (Wang and Dong, 2019). TCA cycle, phosphorylation oxidative, the pentose pathway, and glutaminolysis promote tumor progression (Wang and Dong, 2019). The electron transport chain has been associated with ROS generation where this can act as a secondary messenger in cancer cell survival. Also, this process promotes bioenergetic maintenance for the growth and spread of tumors (Raimondi et al., 2019). *β*-oxidation is related to fatty acids synthesis, which helps in the proliferation and metastatic processes of cancer cells, since they are constituents of the membrane cell, and to develop drug resistance (Ma et al., 2018). Last, the pentose pathway produces ribose-5-phosphate, a substrate that helps in nucleotide synthesis. This route has been related with apoptosis, metastasis, and invasion processes too (Phan et al., 2014).

It can be observed that the action mechanisms of cisplatin and CasIIgly are different, where the latter has effects over related and independent metabolic routes principally in metabolic processes such as carbohydrate and lipid metabolism, thus cancer cells could not have the principal resources for their proliferation and these facts suggest that CasIIgly could be a good alternative for slowing down the processes of metastasis and migration in cancer cells.

**FIGURE 4:**
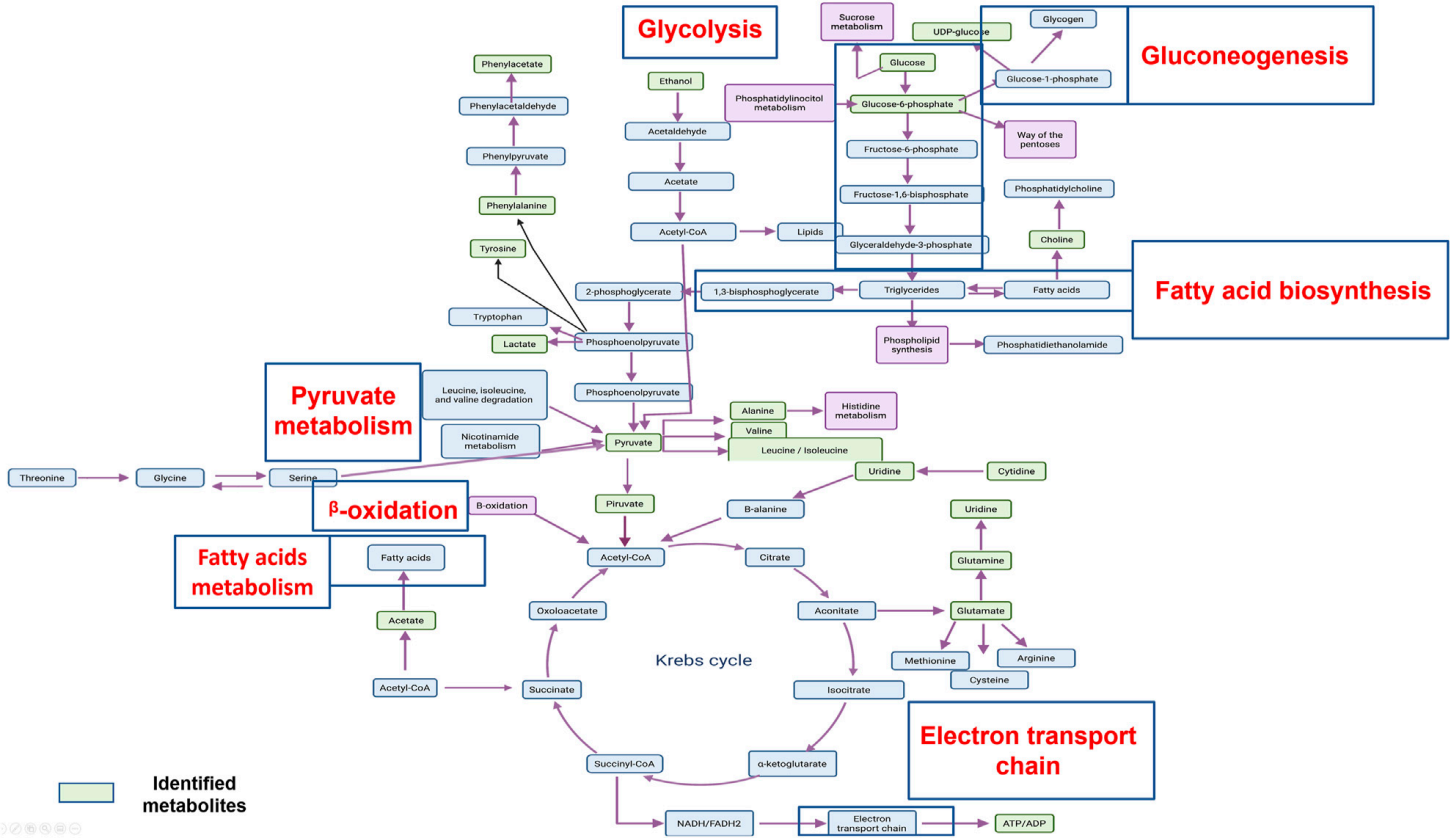
An overview of the important cellular metabolic pathways identified in CasIIgly treatment for both treatment times.

The aforementioned metabolites are implicated in principal metabolic cell pathways and, in particular, in metabolic cancer cell pathways. It is important to consider that most of the metabolic pathways found are related, and some of them are connected, as shown in Figure 4.

In general, we found a smaller number of altered routes in cisplatin-treated cells than CasIIgly-treated cells, which indicates that CasIIgly has a faster effect over cell metabolism, but more experiments with both compounds are required to quanitfy their metabolic affectations over longer periods.

## Conclusion

The Casiopeinas® are one of the first ternary coordination copper compound families for cancer treatment, and this work included the first ^1^H-NMR-based metabolomic analysis applied in this compound type. We highlighted the importance of knowledge of metallodrugs effects on cancer cell metabolism for improving the design of future drugs. Also this paper showed the importance and the versatility of analytical techniques such as ^1^H-NMR, where it can identify and quantify a great variety of metabolites in a highly metastatic cell line.

Cisplatin and CasIIgly treatment had different effects on tumor cell metabolism. Cisplatin mainly affected phospholipid biosynthesis at the times established for this work. CasIIgly acted principally over metabolic processes such as carbohydrate and nucleotide metabolism. A study using longer treatment times could provide us with more information, but with this analysis, it is thought that CasIIgly could have a faster effect than cisplatin and a great impact on the metabolism of MDA-MB-231 cells, which makes it a good alternative for treatment in this type of cancer.

## Data Availability

The original contributions presented in the study are included in the article/Supplementary Material, further inquiries can be directed to the corresponding author.
