# Supplementary material for: 1H -NMR Metabolomics Study of the Effect of Cisplatin and Casiopeina IIgly on MDA-MB-231 Breast Tumor Cells
Source: Front Mol Biosci. 2021 Dec 1;8:742859. doi: 10.3389/fmolb.2021.742859 (PMC8671756; doi:10.3389/fmolb.2021.742859)
Supplement: Supplementary file 1 [file DataSheet1.docx]

**Supplementary Material**

**^1^H -NMR metabolomics study of the effect of cisplatin and Casiopeina IIgly on MDA-MB-231 breast tumor cells**

**Karen Resendiz-Acevedo^1^, Martha E. García-Aguilera^2^, Nuria Esturau-Escofet^2^, Lena Ruiz-Azuara^1^.**

^1^Facultad de Química, Universidad Nacional Autónoma de México, Av. Universidad 3000, Circuito Exterior s/n, CU, C.P. 04510, Ciudad de México, México.

^2^Instituto de Química, Universidad Nacional Autónoma de México, Av. Universidad 3000, Circuito Exterior s/n, CU, C.P. 04510, Ciudad de México, México.

***Correspondence:**Lena Ruiz-Azuara
lenar701@gmail.com

**Supplementary Figure 1.** **(A)** Cell viability average with Cisplatin treatment, (**B)** Cell viability average with CasIIlgly treatment. The error bars represent the standard deviation obtained from the three replicates of each trial.

**Supplementary Table 1.** Metabolites responsible for the separation of the samples at 20 min presented in order of importance (VIP).

| VIP Scores | Bucket (ppm) | Metabolites |
| --- | --- | --- |
| 5.68401 | 1.20-1.18 | Ethanol |
| 4.14227 | 3.68-3.66 | Ethanol, Glucose-6-phosphate, Glycine |
| 4.10365 | 3.38-3.36 | Glucose-6-phosphate, Phenylananine |
| 3.72798 | 2.94-2.92 | Tyrosine |
| 3.69752 | 1.26-1.24 | Etanol |
| 3.50154 | 3.66-3.64 | Ethanol, Glucose-6-phosphate, Glycine |
| 3.36677 | 3.86-3.84 | Adenosine, Alanine, Cytidine, Glucose, Glutamine, Uridine, UDP-glucose |
| 3.3268 | 2.98-2.96 | Tyrosine |
| 3.18899 | 3.70-3.68 | Glucose-6-phosphate, Glutamate, Leucine, Glucose |
| 3.18043 | 1.22-1.20 | Ethanol |
| 3.01687 | 3.00-2.98 | Tyrosine |
| 2.91296 | 1.34-1.32 | Lactate |
| 2.91223 | 3.16-3.14 | Phenylalanine |
| 2.57532 | 3.02-3.00 | Tyrosine |
| 2.51024 | 2.96-2.94 | Tyrosine |
| 2.41446 | 3.72-3.70 | Glucose-6-phosphate, Glutamate, Leucine, Glucose |
| 2.41446 | 3.84-3.82 | Adenosine, Alanine, Cytidine, Glucose, Glutamine, Uridine, UDP-glucose |
| 2.39472 | 0.72-0.70 | NI |
| 2.38012 | 2.24-2.22 | NI |
| 2.32718 | 2.74-2.72 | NI |
| 2.30993 | 3.18-3.16 | Phenylalanine |
| 2.26659 | 1.36-1.34 | Lactate |
| 2.24552 | 1.30-1.28 | Lactate, Isoleucine |
| 2.19737 | 3.88-3.86 | Alanine, Cytidine, Glucose, Glutamine, Uridine, UDP-glucose |
| 2.09504 | 1.94-1.92 | Acetate |
| 2.00361 | 0.14-0.12 | ADP, ATP, NAD, UDP-glucose |
| 1.92345 | 1.50-1.48 | Ethanol, Glucose-6-phosphate, Glycine |
| 1.87821 | 3.24-3.22 | Acetate |
| 1.82006 | 5.92-5.90 | Uridine |
| 1.72335 | 3.50-3.48 | Choline, Glucose, Glycine, Glucose-6-phosphate |
| 1.70024 | 1.18-1.16 | Ethanol, Isoleucine |
| 1.6744 | 3.94-3.92 | Adenosine, Cytidine, Glucose, Glucose-6-phosphate, Uridine, UDP-glucose |
| 1.66036 | 3.42-3.40 | Glucose |
| 1.61768 | 3.48-3.46 | Choline, Glucose, Glycine, Glucose-6-phosphate |
| 1.61699 | 3.04-3.02 | Tyrosine |
| 1.60428 | 3.06-3.04 | Tyrosine |
| 1.55124 | 4.66-4.64 | ATP, Glucose |
| 1.52419 | 4.04-4.02 | Glucose-6-phosphate |

**Supplementary Table 2.** Metabolites responsible for the separation of the samples at 40 min presented in order of importance (VIP).

| VIP Scores | Bucket (ppm) | Metabolites |
| --- | --- | --- |
| 7.32748 | 3.38-3.36 | Glucose-6-phosphate, Phenylananine |
| 5.78828 | 1.26-1.24 | Ethanol |
| 4.72643 | 3.70-3.68 | Glucose-6-phosphate, Glutamate, Leucine, Glucose |
| 4.28473 | 1.34-1.32 | Lactate |
| 3.94671 | 3.68-3.66 | Ethanol, Glucose-6-phosphate, Glycine |
| 3.74043 | 1.94-1.92 | Acetate |
| 3.34251 | 3.72-3.70 | Glucose-6-phosphate, Glutamate, Leucine, Glucose |
| 3.21289 | 3.02-3.00 | Tyrosine |
| 2.92317 | 3.66-3.64 | Ethanol, Glucose-6-phosphate, Glycine |
| 2.90059 | 0.72-0.70 | NI |
| 2.83577 | 1.20-1.18 | Ethanol |
| 2.68583 | 0.14-0.12 | ADP, ATP, NAD, UDP-glucose |
| 2.39979 | 5.92-5.90 | Uridine |
| 2.38602 | 1.36-1.34 | Lactate |
| 2.38092 | 3.86-3.84 | Adenosine, Alanine, Cytidine, Glucose, Glutamine, Uridine, UDP-glucose |
| 2.36098 | 3.00-2.98 | Tyrosine |
| 2.30014 | 1.28-1.26 | Ethanol |
| 2.23601 | 2.96-2.94 | Tyrosine |
| 2.20659 | 3.78-3.76 | Glucose-6-phosphate, Glutamate, Leucine, Glucose |
| 2.20437 | 2.94-2.92 | Tyrosine |
| 2.1546 | 3.16-3.14 | Phenylalanine |
| 2.09377 | 3.88-3.86 | Alanine, Cytidine, Glucose, Glutamine, Uridine, UDP-glucose |
| 2.03841 | 1.24-1.22 | Ethanol |
| 2.03569 | 2.38-2.36 | Glutamate, Valine |
| 1.93969 | 3.84-3.82 | Adenosine, Alanine, Cytidine, Glucose, Glutamine, Uridine, UDP-glucose |
| 1.90869 | 3.90-3.88 | Alanine, Cytidine, Glucose, Glutamine, Uridine, UDP-glucose |
| 1.88817 | 3.74-3.72 | Glucose-6-phosphate, Glutamate, Leucine, Glucose |
| 1.83076 | 1.30-1.28 | Lactate, Isoleucine |
| 1.79324 | 3.06-3.04 | Ethanol |
| 1.79324 | 7.86-7.84 | UDP-glucosa |
| 1.75324 | 3.18-3.16 | Uridine |
| 1.70766 | 3.58-3.56 | Glutamate, Glutamine |
| 1.68671 | 0.94-0.92 | Tyrosine |
| 1.67109 | 2.98-2.96 | Cytidine |
| 1.65036 | 8.02-8.00 | Glutamate, Valine |
| 1.59689 | 1.22-1.20 | ATP, UDP-glucose |
| 1.55755 | 4.14-4.12 | Choline, Cytidine, Lactate |
| 1.54074 | 0.96-0.94 | Leucine |
| 1.53451 | 1.68-1.66 | NI |
| 1.51012 | 6.08-6.06 | Adenosine |
| 1.50756 | 1.76-1.74 | Leucine |

***NI:** the metabolites corresponding to this bucket weren´t identify.

**Supplementary Table 3.** Concentration and standard deviation of metabolites responsible for the samples separation to 20 and 40 min for cells without treatment.

|  | Concentration mean (μM) | |
| --- | --- | --- |
| Metabolite | **No treatment**  **20 min** | **No treatment**  **40 min** |
| ADP | 0.003 ± 0.003 | 0.004 ± 0.003 |
| ATP | 0.002 ± 0.002 | 0.004 ± 0.003 |
| Acetate | 0.024 ± 0.027 | 0.015 ± 0.013 |
| Adenosine | 0.001 ± 0.000 | 0.003 ± 0.002 |
| Alanine | 0.016 ± 0.014 | 0.024 ± 0.011 |
| Choline | 0.001 ± 0.001 | 0.030 ± 0.007 |
| Glucose | 0.026 ± 0.032 | 0.027 ± 0.022 |
| Glucose-6-phosphate | 0.022 ± 0.026 | 0.014 ± 0.006 |
| Glutamate | 0.038 ± 0.034 | 0.058 ± 0.015 |
| Glutamine | 0.007 ± 0.007 | 0.012 ± 0.007 |
| Glycine | 0.018 ± 0.016 | 0.030 ± 0.007 |
| Isoleucine | 0.005 ± 0.004 | 0.011 ± 0.007 |
| Lactate | 0.022 ± 0.020 | 0.035 ± 0.023 |
| Leucine | 0.007 ± 0.006 | 0.019 ± 0.012 |
| NAD+ | 0.011 ± 0.010 | 0.010 ± 0.002 |
| Phenylalanine | 0.003 ± 0.002 | 0.005 ± 0.003 |
| Tyrosine | 0.003 ± 0.003 | 0.007 ± 0.005 |
| Uridine | 0.009 ± 0.010 | 0.021 ± 0.013 |
| Valine | 0.009 ± 0.002 | 0.007 ± 0.005 |

**Supplementary Table 4.** Concentration and standard deviation of metabolites responsible for the samples separation to 20 and 40 min for Cisplatin treatment.

|  | | Concentration mean (μM) | |
| --- | --- | --- | --- |
| Metabolite | | **Cisplatin 20 min** | **Cisplatin 40 min** |
| Acetate | | 0.096 ± 0.014 | 0.032 ± 0.008 |
| Adenosine | | 0.016 ±0.001 | 0.008 ± 0.005 |
| ADP | | 0.005 ± 0.002 | 0.003 ± 0.003 |
| Alanine | | 0.031 ± 0.003 | 0.026 ± 0.006 |
| ATP | | 0.004 ± 0.002 | 0.003 ± 0.002 |
| Coline | | 0.004 ± 0.001 | 0.024 ± 0.009 |
| Cytidine | | 0.005 ± 0.000 | 0.008 ± 0.005 |
| Glicine | | 0.014 ± 0.004 | 0.024 ± 0.009 |
| Glucose | | 0.013 ± 0.001 | 0.038 ± 0.015 |
| Glucose-6-phosphate | | 0.017 ± 0.004 | 0.024 ± 0.013 |
| Glutamate | | 0.063 ± 0.014 | 0.052 ± 0.018 |
| Glutamine | 0.046 ± 0.016 | 0.009 ± 0.003 |  |
| Isoleucine | | 0.033 ± 0.012 | 0.006 ± 0.000 |
| Lactate | | 0.010 ± 0.005 | 0.048 ± 0.005 |
| Leucine | | 0.005 ± 0.001 | 0.010 ± 0.001 |
| NAD+ | | 0.024 ± 0.003 | 0.011 ± 0.005 |
| Phenylacetate | | 0.022 ± 0.001 | 0.021 ± 0.009 |
| Phenylananine | | 0.004 ± 0.004 | 0.003 ± 0.001 |
| Piruvate | | 0.004 ± 0.002 | 0.006 ± 0.001 |
| Succinate | | 0.004 ± 0.001 | 0.010 ± 0.014 |
| Tirosine | | 0.003 ± 0.001 | 0.003 ± 0.01 |
| UDP-glucose | | 0.032 ± 0.002 | 0 ± 0.000 |
| Uridine | | 0.004 ± 0.003 | 0.015 ± 0.007 |
| Valine | | 0.027 ± 0.001 | 0.008 ± 0.001 |

**Supplementary Table 5.** Concentration and standard deviation of metabolites responsible for the samples separation to 20 and 40 min for CasIIlgly treatment.

|  | Concentration mean (μM) | |
| --- | --- | --- |
| Metabolite | **CasIIgly 20 min** | **CasIIgly 40 min** |
| ADP | 0.004 ± 0.002 | 0.000 ± 0.000 |
| ATP | 0.005 ± 0.001 | 0.010 ± 0.014 |
| Acetate | 0.039 ± 0.010 | 0.020 ± 0.015 |
| Adenosine | 0.003 ± 0.001 | 0 ± 0.000 |
| Alanine | 0.028 ± 0.006 | 0.021 ± 0.014 |
| Choline | 0.001 ± 0.001 | 0.001 ± 0.001 |
| Cytidine | 0.008 ± 0.005 | 0.001 ± 0.003 |
| Ethanol | 0.004 ± 0.001 | 0.017 ± 0.008 |
| Glucose | 0.058 ± 0.015 | 0.042 ± 0.031 |
| Glucose-6-phosphate | 0.034 ± 0.009 | 0.026 ± 0.016 |
| Glutamate | 0.036 ± 0.004 | 0.043 ± 0.004 |
| Glutamine | 0.010 ± 0.003 | 0.008 ± 0.003 |
| Glycine | 0.020 ± 0.004 | 0.018 ± 0.007 |
| Isoleucine | 0.008 ± 0.001 | 0.006 ± 0.005 |
| Lactate | 0.036 ± 0.008 | 0.028 ± 0.021 |
| Leucine | 0.012 ± 0.002 | 0.009 ± 0.005 |
| NAD+ | 0.006 ± 0.003 | 0.007 ± 0.005 |
| Phenylalanine | 0.004 ± 0.001 | 0.004 ± 0.003 |
| Tyrosine | 0.006 ± 0.002 | 0.005 ± 0.001 |
| Uridine | 0.012 ± 0.003 | 0.021 ± 0.013 |
| Valine | 0.009 ± 0.002 | 0.007 ± 0.005 |

**Supplementary Table 6.** Metabolite sets enrichment without treatment at 20 and 40 min.

***Total** is the total number of compounds in the pathway.; Hits is the matched number of metabolites from the uploaded data; **Statistic Q** is the average of the Q statistics for each metabolite in the set; **Raw p** is the original p-value calculated from enrichment analysis; **Holm p** is the p value adjusted by Holm-Bonferroni method; **FDR** is the p value adjusted using False Discovery Rate and Impact is the pathway impact value calculated from topology analysis.

**Supplementary Table 7.** Metabolite sets enrichment for Cisplatin treatment at 20 and 40 min.

***Total** is the total number of compounds in the pathway.; Hits is the matched number of metabolites from the uploaded data; **Statistic Q** is the average of the Q statistics for each metabolite in the set; **Raw p** is the original p-value calculated from enrichment analysis; **Holm p** is the p value adjusted by Holm-Bonferroni method; **FDR** is the p value adjusted using False Discovery Rate and Impact is the pathway impact value calculated from topology analysis.

**Supplementary Table 8.** Metabolite sets enrichment for CasIIgly treatment at 20 and 40 min.

***Total** is the total number of compounds in the pathway. Hits is the matched number of metabolites from the uploaded data; **Statistic Q** is the average of the Q statistics for each metabolite in the set; **Raw p** is the original p-value calculated from enrichment analysis; **Holm p** is the p value adjusted by Holm-Bonferroni method; **FDR** is the p value adjusted using False Discovery Rate and Impact is the pathway impact value calculated from topology analysis.
